# Supplementary material for: Exploring the Role of Different Cell-Death-Related Genes in Sepsis Diagnosis Using a Machine Learning Algorithm
Source: Int J Mol Sci. 2023 Sep 29;24(19):14720. doi: 10.3390/ijms241914720 (PMC10572834; doi:10.3390/ijms241914720)
Supplement: Supplementary file 1 [file ijms-24-14720-s001.zip › ijms-2561747-supplementary.pdf]

Supplementary material

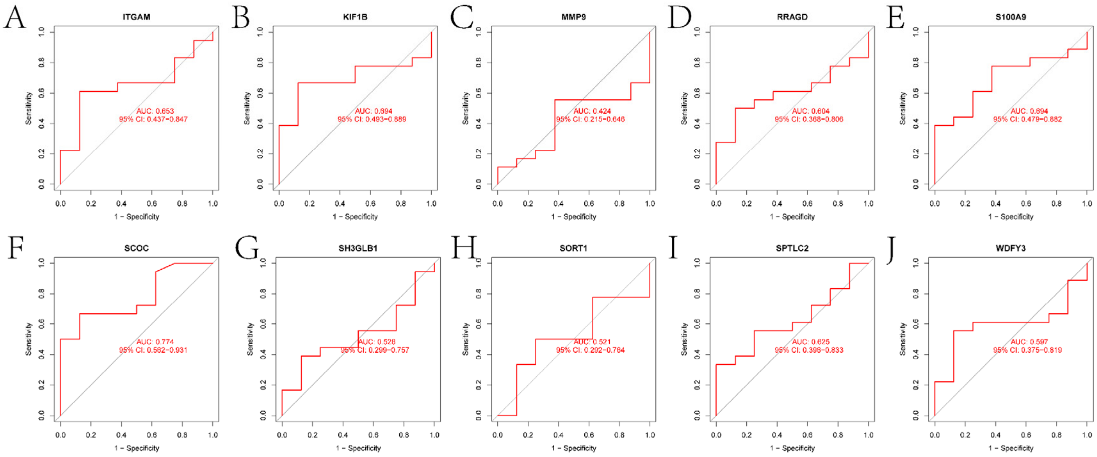

Figure S1. The verification results of diagnosis-related genes in the internal test set.

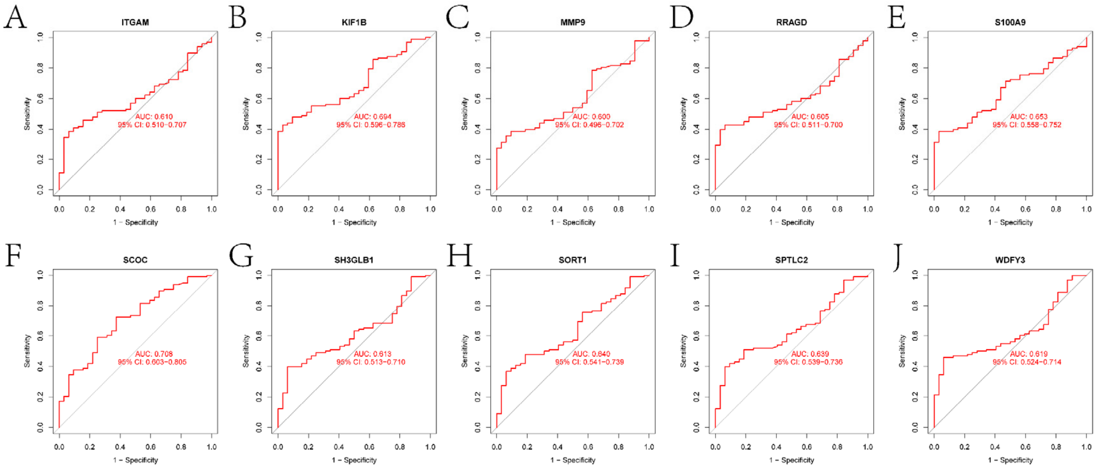

Figure S2. The verification results of diagnosis-related genes in external test set.

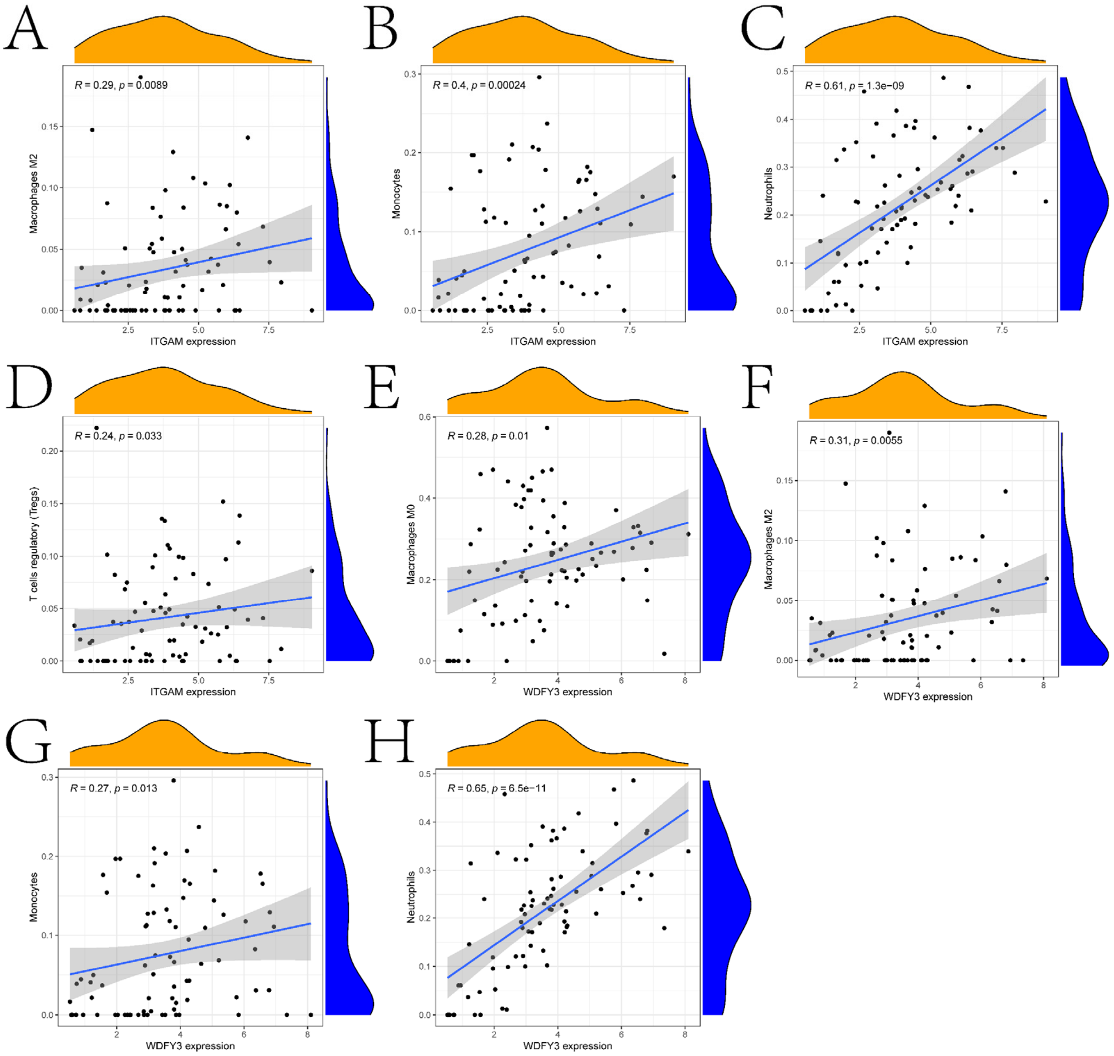

Figure S3. Correlation analysis between the last five diagnosis-related genes and immune cells. A-D gives the scatter diagram of the correlation between ITGAM and immune cells. E-I gives the scatter plot of correlation between WDFY3 and immune cells.

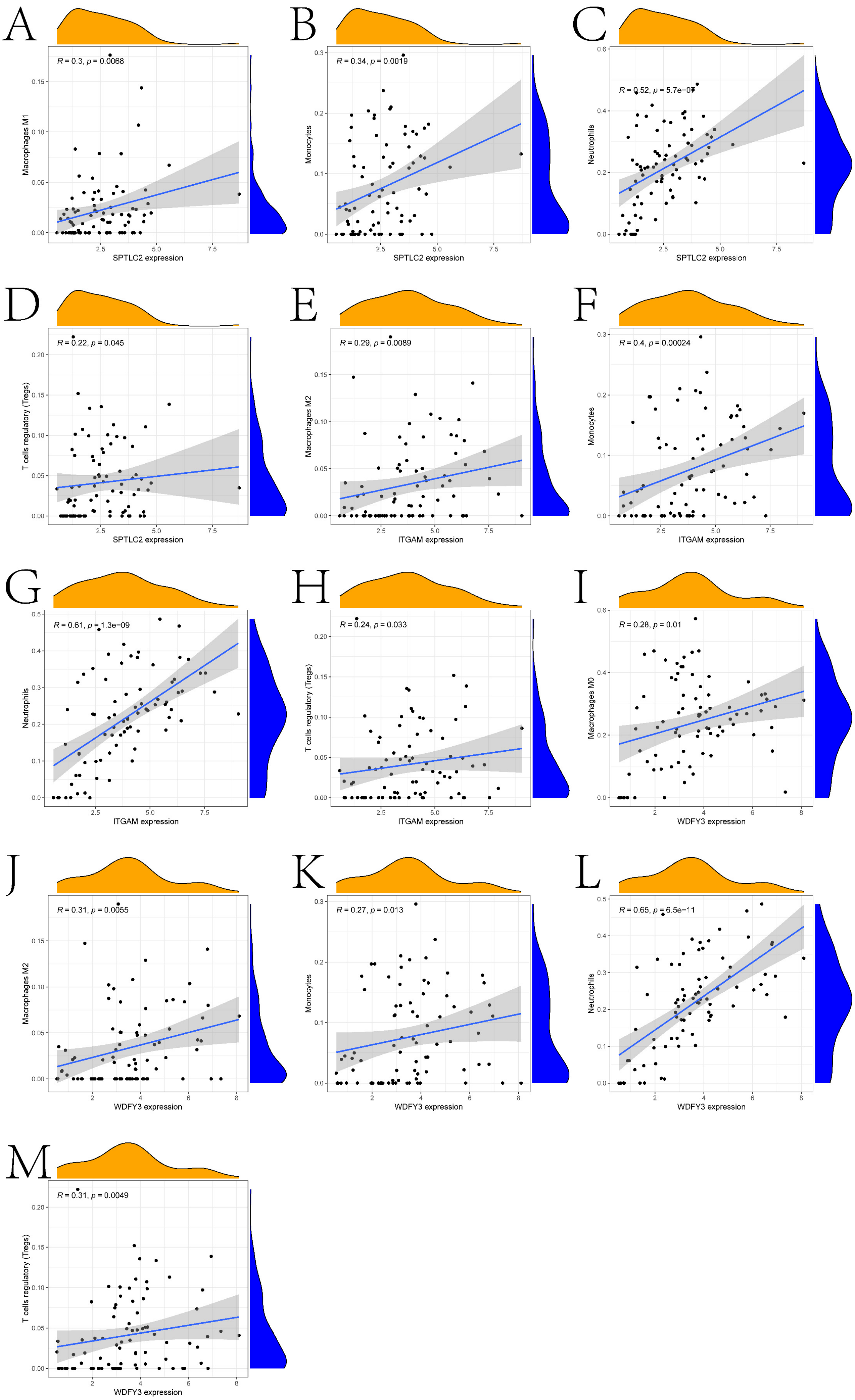
A
R = 0.3, p = 0.0068
Macrophages M1
SPTLC2 expression
B
R = 0.34, p = 0.0019
Monocytes
SPTLC2 expression
C
R = 0.52, p = 5.7e−07
Neutrophils
SPTLC2 expression
D
R = 0.22, p = 0.045
T cells regulatory (Tregs)
SPTLC2 expression
E
R = 0.29, p = 0.0089
Macrophages M2
ITGAM expression
F
R = 0.4, p = 0.00024
Monocytes
ITGAM expression
G
R = 0.61, p = 1.3e−09
Neutrophils
ITGAM expression
H
R = 0.24, p = 0.033
T cells regulatory (Tregs)
ITGAM expression
I
R = 0.28, p = 0.01
Macrophages M0
WDFY3 expression
J
R = 0.31, p = 0.0055
Macrophages M2
WDFY3 expression
K
R = 0.27, p = 0.013
Monocytes
WDFY3 expression
L
R = 0.65, p = 6.5e−11
Neutrophils
WDFY3 expression
M
R = 0.31, p = 0.0049
T cells regulatory (Tregs)
WDFY3 expression

Figure S4. A-D gives the scatter plot of the correlation between SPTLC2 and immune cells. E-H gives the scatter plot of correlation between ITGAM and immune cells. I-M gives the scatter plot of correlation between WDFY3 and immune cells.

Table S1. Demographic information for the Sepsis group and the Control group.

Parameters	Control	Sepsis	*P*
**Gender**			0.614
Male	4	3	
Female	4	5	
**Ages**			N
≥60	1	5	
18-60	7	3	
≤18	0	0	
**Infection Source**			N
Lung	0	4	
Urinary tract	0	1	
Skin or Soft tissues	0	0	
Other	0	3	
**Comorbidities**			0.001
Yes	0	7	
No	8	1	
**ΔSOFA Scores**			0.001
0-2	8	0	
≥2	0	8	
**Lactate levels[a] (mmol/L)**	0.81±0.33	3.65±2.45	0.001

[a] Lactate levels: Mean ± standard deviation, measurement time point: Sepsis group: within 24h after diagnosis of sepsis; Control group: the day of physical examination.
